# Inflammatory pseudotumor of the spleen: A case report and review of published cases

**DOI:** 10.3892/ol.2013.1286

**Published:** 2013-04-03

**Authors:** ZHEN-HAI MA, XIAO-FENG TIAN, JINHUI MA, YONG-FU ZHAO

**Affiliations:** Department of General Surgery, The Second Affiliated Hospital of Dalian Medical University, Dalian, Liaoning 116027, P.R. China

**Keywords:** inflammatory pseudotumor, spleen, benign tumor, misdiagnosis

## Abstract

Inflammatory pseudotumor of the spleen (IPTS) is an extremely rare condition. To the best of our knowledge, only ∼113 cases have been reported in the literature since the first 2 cases were reported in 1984. The present study reports the case of an IPTS in a 72-year-old male patient. The splenic tumor was identified incidentally 1 year prior to the patient being admitted to the Second Affiliated Hospital of Dalian Medical University (Dailan, China). There were no specific clinical symptoms. The initial diagnosis was of splenic lymphoma based on the pre-operative radiological findings. However, the patient underwent a splenectomy and the final pathological diagnosis of IPTS was declared. The present study also highlighted the difficulty of forming accurate pre-operative diagnoses, even when using modern imaging techniques. A partial resection of the spleen or splenectomy was considered to be the required treatment to form a definitive diagnosis and exclude malignancy. The prognosis of IPTS is generally considered to be favorable following splenectomy. The clinical and pathological features of previously reported cases are also briefly reviewed in the present study to aid in improving the accuracy of the diagnosis of this rare disease.

## Introduction

Splenic tumors are relatively rare and difficult to diagnose prior to surgery. Splenic neoplasms include hemangioma, lymphangioma, hamartoma, hemangiosarcoma, malignant lymphoma and metastatic carcinoma. Inflammatory pseudotumors of the spleen (IPTSs) are extremely rare and frequently misdiagnosed as malignant or as other tumors prior to surgery (1,2). IPTSs are benign entities of unknown etiology and pathogenesis that have been described in only a few cases in the literature (3). The present study reports a new case and reviews the clinicopathological features, diagnosis, treatment and prognosis of the previously reported cases of IPTS. Informed consent was obtained from the patient’s family.

## Case report

**Figure 1:**
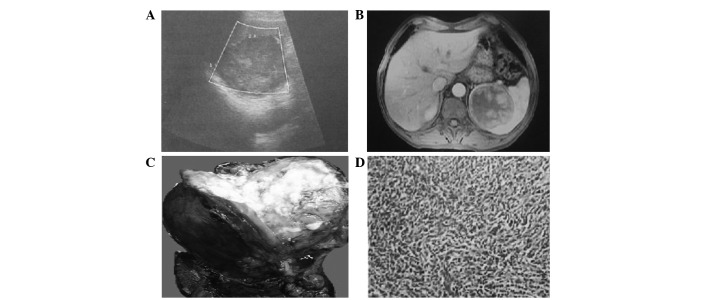
(A) Abdominal ultrasonography showing a single splenic mass 5.5 cm in diameter. (B) MRI showing the lesion in the upper spleen. A T1 dynamic delay phase study demonstrated enhancement of the periphery and inhomogeneous enhancement in the tumor center. (C) The resected spleen weighed 385 g and the tumor size was 7.8×6.5×5.5 cm. The tumor was circumscribed, but not encapsulated, and contained a large amount of tan-white necrotic tissue in the center. (D) Low-magnification photomicrograph of normal spleen showing mixed inflammatory infiltration, a well-defined fibrous capsule and adjacent splenic tissue with a resemblance to granulation tissue (hematoxylin and eosin staining, magnification ×100). MRI, magnetic resonance imaging.

A 72-year-old male was admitted to the Department of General Surgery at the Second Affiliated Hospital of Dalian Medical University (Dailan, China) due to a tumor of the spleen that had been identified incidentally 1 year previously and which had grown in diameter over a 15-day period prior to the admittance. The splenic mass was detected in a routine ultrasound scan 1 year prior to admittance. At the time of identification, the diameter was 5.5 cm (Fig. 1A), while at the time of admittance, 1 year later, the diameter was 7.7 cm. The patient remained asymptomatic. A physical examination revealed that the patient had no fever, or abdominal pain and distension. The patient’s abdomen was flat, with no tenderness. The biochemical and hematological investigations were all within the normal ranges. Magnetic resonance imaging (MRI; Fig. 1B) and computed tomography (CT) scans of the abdomen were performed and confirmed the presence of a mass within the spleen demonstrating diffuse heterogeneous enhancement. The tumor was suspected to be a splenic lymphoma or another type of malignant tumor. Consequently, the decision was made to proceed with surgery and the patient underwent a splenectomy.

On entering the abdominal cavity, the splenic tumor was visible and occupied the majority of the spleen, therefore, the patient underwent a splenectomy. The resected spleen weighed 385 g and the tumor size was 7.8×6.5×5.5 cm. When the spleen was placed into a pan, it was noted that the tumor was circumscribed, but not encapsulated, and contained a large amount of tan-white, necrotic tissue in the center (Fig. 1C).

On histological examination, a large, irregularly-shaped necrotic focus was observed in the center, with a marked area of inflammatory infiltration. This was composed of an admixture of inflammatory cellular elements, predominantly plasma cells and lymphocytes with hyalinization, fibrosis, lymph follicles and multinuclear giant cells (Fig. 1D). The final pathological diagnosis was of IPTS.

The patient thus far remains alive and asymptomatic at 4 months subsequent to surgery.

## Discussion

Inflammatory pseudotumors occur in a variety of organs and locations, including the orbit of the eye, the respiratory tract, the gastrointestinal tract and the liver. However, IPTSs are extremely rare lesions that are usually located incidentally (1,4). The incidence of benign splenic tumors is 0.007% among all subjects on whom surgeries or autopsies are performed. Consequently, the incidence of IPTS is much lower than this (5). To the best of our knowledge, since Cotelingam and Jaffe (6) first reported 2 cases of IPTS in 1984, only 114 cases, including the present case, have been reported in the literature.

Based on these studies, it appears that IPTS usually affects middle- and advanced-aged adults. Only 4 cases have been reported in children (7–10). However, there is controversy (11) over the association between IPTS and gender. The majority of studies have suggested that women are more frequently affected (12). A review of the 114 reported cases of IPTS was performed, which showed that there were 48 cases that listed the gender of the patients. Among these cases there were 18 males and 30 females, with a 5:3 female predominance. The patients ranged in age from 6 to 81 years, with a median age of 47.2 years.

The pathogenesis of IPTS is a topic of debate and several possible causes have been reported. Bacterial infection, neoplastic processes, vascular causes and immunological derangement have all been proposed as factors. Certain cases have been reported to be Epstein-Barr virus-positive inflammatory pseudotumors. However, the real pathogenesis remains unknown (1,13–16).

IPTSs often present diagnostic difficulties, since they lack characteristic clinical manifestations and the symptoms are extremely diverse. The majority of lesions (n=76, 66.7%) were detected incidentally during routine physical check-ups or elective abdominal imaging studies. Among the patients exhibiting symptoms, upper abdominal pain or discomfort (n=23, 60.5%, 23/38) was the predominant symptom. Fever and splenomegaly were also present in certain cases (17).

When a lesion occurs as a primary splenic tumor, lymphoma is usually clinically suspected (18), as occurred in the present patient. The laboratory data of a number cases showed no evidence of any abnormalities. Although imaging examinations have improved significantly in the past 2 decades, they have been unable to provide conclusive results. Only pathological and immunohistochemical studies following splenectomy have enabled any definitive diagnoses.

Ultrasound, MRI and CT scans are able to provide a certain amount of evidence for diagnosis. However, these findings are not specific enough to differentiate this type of lesion from other neoplasms. Ultrasonography may reveal a partially calcified, well-defined echogenic mass or hypoechoic discrete lesion, consistent with the findings in the present case. CT scans usually show a low-density mass in the non-enhanced and enhanced modes. MRI may reveal a well-defined mass in a superior manner to a CT scan, which is reported to be isointense on T1-weighted images, and with either an increased or decreased signal intensity on T2-weighted images, with respect to the surrounding normal spleen (1,2,19).

Performing a core biopsy is a useful method for diagnosing hematological and non-hematological splenic lesions that may be used to distinguish IPTSs from other lesions in its differential diagnosis (16,20). However, a core biopsy is not recommended for splenic masses due to the uncertainty of detecting the disease, the risk of metastases if the mass is a malignant neoplasm and the potential hemorrhagic complications of the procedure. Therefore, a histological examination of the resected specimens is the gold standard for diagnosing tumors of the spleen.

The typical macroscopic appearance of an IPTS lesion is that of a well-circumscribed, non-encapsulated mass with a large amount of tan-white, necrotic tissue in the center. The cellular composition of IPTSs may be remarkably heterogeneous. The IPTS mass may resemble granulation tissue, while normal lymphocytes and plasma cells are constant features, although variable in mixture and number. Neutrophilic and eosinophilic leukocytes are also present in certain cases. This may raise the possiblity of a lymphoreticular malignancy, thus requiring immunohistological studies for a definitive diagnosis in certain cases (14).

When an IPTS is identified, it must first be differentiated from lymphatic neoplasma, particularly malignant lymphoma. A diagnosis of malignant lymphoma was initially made in a number of the IPTS cases reported in the literature, as occurred in the present case prior to surgery. The differential diagnosis for IPTS also considers other lesions, including hamartomas, hemangiomas, hemangioendotheliomas, angiosarcomas, infectious granulomatous processes and sarcoidosis (4–7,11–15,21,22).

As an IPTS lesion is benign without the risk of malignant transformation, the question arises whether an asymptomatic patient with IPTS should undergo a surgical procedure if the lesion is detected incidentally. At present there is no sensitive and specific method for diagnosing IPTS without a tissue sample, and since certain lesions that resemble IPTS are malignant in nature, we propose that it is prudent to operate even if IPTS is suspected. If the lesion is not too large, a splenectomy is unnecessary and partial splenic resection, using laparoscopic or open surgery, is a relatively suitable surgical approach. Recently, there have been several studies concerned with the use of laparoscopic spleen surgery for IPTS (23–25).

According to the previously published cases, the prognosis of IPTS has generally been considered to be favorable following splenectomy, although there is minimal data available on the follow-up of these patients. There have been no reports of metastatic disease, local invasion or recurrence. However, careful follow-up is necessary, since certain patients with inflammatory pseudotumors of the liver have been reported to have succumbed to the disease (1,26,27).

To conclude, IPTS is an extremely rare condition. To the best of our knowledge, only 114 cases have been reported in the literature, including the present case. Establishing a pre-operative diagnosis of IPTS is often difficult and since certain lesions that resemble IPTS are malignant in nature, we suggest that it is prudent to operate even if IPTS is suspected. Only splenectomy and histopathological study of the specimen enable a definitive diagnosis and the consequent treatment of this disease, therefore splenectomy is diagnostic and curative. The prognosis of IPTS has generally been considered to be favorable following splenectomy.
